# Heating Performance and Electrical Properties of Cement Composites Using Conductive Films Coated with Multi-Walled Carbon Nanotubes

**DOI:** 10.3390/ma18122773

**Published:** 2025-06-12

**Authors:** Jong-Gun Park, Dong-Ju Seo, Chang-Ho Yun, Tae-Hyoung Kim, Ki-Chang Song, Gwang-Hee Heo

**Affiliations:** 1 Public Safety Research Center (PSRC), Konyang University, 121, Daehak-ro, Nonsan-si 32992, Republic of Korea; 2 Department of Disaster and Safety Engineering, Konyang University, 121, Daehak-ro, Nonsan-si 32992, Republic of Korea; ws9500@naver.com (D.-J.S.); forchyun99@korea.kr (C.-H.Y.); 3 Department of Biomedical Materials, Konyang University, Daejeon 35365, Republic of Korea; 20856503@konyang.ac.kr (T.-H.K.); songkc@konyang.ac.kr (K.-C.S.); 4 Department of International Civil and Plant Engineering, Konyang University, 121, Daehak-ro, Nonsan-si 32992, Republic of Korea; heo@konyang.ac.kr

**Keywords:** multi-walled carbon nanotubes (MWCNTs), polyethylene terephthalate (PET), cement composites, heating performance, electrical properties

## Abstract

In this study, multi-walled carbon nanotubes (MWCNTs) were uniformly applied to polyethylene terephthalate (PET) film using a bar-coating method to fabricate conductive thin films, and their transmittance, surface morphology, and effects on the heating and electrical properties of cement composites were analyzed. The experimental parameters considered were the mixing method, MWCNT concentrations, use or absence of coating films, applied voltages, and electrode spacings. Considering these parameters, the cement composites were divided into a total of four groups and then fabricated. Group 1 is a method for fabricating plain cement composites (PCCs), while Group 2 is a method for fabricating PCC using only MWCNT-coated films. Group 3 is a method for fabricating PCC by adding only MWCNT dispersion, and finally, Group 4 is a method for fabricating PCC using both MWCNT dispersion and MWCNT-coated films. Furthermore, field emission scanning electron microscope (FE-SEM) image analysis confirmed that MWCNT were evenly distributed across the entire front surface of the PET film and formed a dense network structure. The experimental results of cement composites using these showed that when both MWCNT dispersion and MWCNT-coated films were used, the electrical resistance was significantly reduced and the heating performance was improved. In particular, when the electrode spacing was 40 mm and the applied voltage was 30 V, the MDCF-0.75 specimen exhibited the highest heating performance and the lowest electrical resistance.

## 1. Introduction

Cement composites are representative composite materials composed of cement, aggregate, and water, and have long been the most widely used construction materials in the world due to their excellent mechanical properties, durability, and economic efficiency [1,2]. Since the latter half of the 20th century, particularly, research has been actively conducted to impart electrical properties to cement composites by mixing conductive materials [3,4,5].

Conventional cement composites have traditionally been recognized as insulators and are generally known to have very high electrical resistance and little electrical conductivity. However, by adding conductive materials, a continuous network can be formed, resulting in conductive cement composites capable of conducting electricity. When electrodes are connected to these complexes and voltage is applied, current flows and resistive heating occurs, increasing the surface temperature, which can then be utilized as a new type of cement complex. These properties have potential applications in various fields such as deicing and snow-melting on pavements in winter, improvement of the living environment, and efficiency of the construction process [6,7,8,9,10,11,12].

Carbon-based conductive materials include carbon nanotubes, carbon black, graphite powder, graphene, and carbon fiber, which are widely used in cement composites. Especially, carbon nanotubes (CNTs) are classified into single-walled carbon nanotubes (SWCNTs), double-walled carbon nanotubes (DWCNTs), and multi-walled carbon nanotubes (MWCNTs). Nanomaterials have recently attracted the attention of many researchers because they can effectively improve the mechanical, electrical, and thermal performance of materials when mixed with construction materials of cement composites [13,14,15,16,17,18,19,20]. Mixing nanomaterials with existing construction materials has great potential to be utilized as multifunctional cement composites [21,22,23]. Nanomaterials with particle sizes ranging from 0.1 to 100 nm have attracted much attention in various fields due to their high surface area per unit weight and excellent properties [24,25,26]. In particular, CNTs have electrical conductivity similar to that of metallic materials and a very high aspect ratio (length/diameter) of over 1000, so they can form efficient conductive paths within a composite even in relatively small quantities. In consideration of these properties, it is necessary to develop multifunctional cement composites that can effectively melt snow and ice while maintaining the strength and durability of the materials to solve the problem of icy roads and black ice on pavements during winter. Research on the applicability of cement composites containing MWCNT as self-heating elements is still lacking. Dispersed MWCNT forms a uniform conductive network that enhances the overall electrical conductivity, whereas the MWCNT-coated film functions as a surface heating element. The combined use of the two results in a synergistic effect that notably enhances heating performance compared to their individual applications.

In previous studies, the heating performance and electrical properties of cement composites using MWCNT were analyzed. In general, the method of incorporating MWCNT in cement composites has been widely employed, in which MWCNT are evenly dispersed in a solution and then mixed with cement [27,28]. Gupta et al. [29] proposed a novel method of coating carbon nanotube-based ink on the surface of aggregates, which resulted in the creation of self-sensing concrete specimens that were conductive and exhibited electrical properties that changed depending on damage. In addition, an electrical impedance tomography algorithm was implemented to generate a resistivity map that could identify the location and severity of damage, and the effectiveness of this approach was verified through various specimens. Konsta and Aza [30] analyzed the electrical properties and piezoresistive sensitivity of cement composites containing CNT and CNF (carbon nanotube fiber). As a result, the cement composites formed using both CNT and CNF at a concentration of 0.1 wt.% relative to the cement weight exhibited the highest piezoresistive sensitivity. Lee et al. [31] studied the heating performance of CNT-based cement composites. In the study, SWCNT and MWCNT were used at concentrations of 0.0625 wt.% and 0.125 wt.% (based on cement weight), respectively, and voltages 50 V and 100 V were applied. As a result, the composite with a total CNT concentration of 0.125 wt.% showed superior heating performance compared to the composite with a total CNT concentration of 0.0625 wt.%. Zhang and Li [32] studied the road deicing performance of cement composites containing MWCNT. According to the results, a composite with a thermal conductivity of 2.83 W/m·K was formed when MWCNTs were mixed at a concentration of 2.0 wt.% based on the weight of cement. Therefore, it turned out to be possible to melt ice formed on pavement using MWCNT-cement composites. Meanwhile, polyethylene terephthalate (PET) film is used in various industrial fields due to its excellent mechanical properties and transparency, as well as its excellent chemical resistance and heat resistance. Lately, research has been conducted on analyzing the physical, thermal, and electrical properties of CNT using nanomaterial coating films, and on the possibility of fabricating and applying nanomaterial films and composites based on the analyses.

Huang et al. [33] studied the thermal conductivity according to the arrangement method of CNT films. The study found that thermal conductivity was higher when CNTs were aligned in the same direction at the same CNT concentration. Hone et al. [34] made SWCNT film through filtration and adsorption using a magnetic field and studied the heating performance. The SWCNT film fabricated at this time showed a thermal conductivity of 200 W/m·K, similar to graphite and highly crystalline diamond. Jang and Park [35] fabricated a 25 mm × 25 mm film using dispersed CNT. In the study, they analyzed the electrical conductivity and the heating performance of the films after changing the CNT concentration and film thickness. The results showed that, as the CNT concentration increased, the CNT network became denser and the electrical resistance decreased. Kim et al. [36] made CNT films measuring 10 mm × 10 mm using electrostatic spray deposition without using a binder, and analyzed the electrical conductivity. The fabricated CNT films exhibited excellent electrical conductivity. Pham et al. [37] analyzed the change in electrical resistance of conductive CNT and polymer films under tensile strain. The results confirmed that, as the tensile strain increased, the density of the conductive CNT network decreased and the distance between the tubes increased, which pushed the electrical resistance upwards.

Therefore, the present study aims to develop a novel method to improve the heating performance and electrical properties of cement composites using conductive films coated with MWCNT. To this end, a coating solution was prepared by mixing MWCNT with water-dispersed polyurethane at various mixing ratios, and this was applied to the entire surface of PET film to form an MWCNT-coated conductive thin film. The transmittance and surface morphology of the coating films according to the incorporation ratio of various MWCNT were analyzed, and the effects of using these films on the heating performance and electrical properties of the cement composites were investigated. The experimental parameters considered were mixing method, MWCNT concentrations, use or absence of coating films, applied voltages, and electrode spacings. These were also compared and examined with plain cement composites (PCCs). Furthermore, the microstructure was analyzed using a field emission scanning electron microscope (FE-SEM).

## 2. Experimental Program

### 2.1. Materials

The cement used in this study was ordinary Portland cement (OPC) from Company S (Ssangyong Cement, Seoul, Republic of Korea), with a specific gravity of 3.13 and a fineness of 3860 cm^2^/g [38]. In addition, the physical properties of the standard sand used in this study are shown in Table 1. The standard sand produced from Hyangho-ri, Jumunjin-eup, Gangneung-si, and Gangwon-do was used as fine aggregate to create homogeneous cement composites. The specific gravity and absorption rate of the saturated surface-dried fine aggregate were 2.65 and 0.1%, respectively. The fine aggregate used was a standard sand containing 98% or more of silicon dioxide (SiO_2_) and with a maximum particle size of no more than 2.0 mm, as specified in KS L ISO 679 [39]. The film thickness of the transparent and flexible PET film used in this study was approximately 100 μm, and its main properties are shown in Table 2.

**Figure 1:**
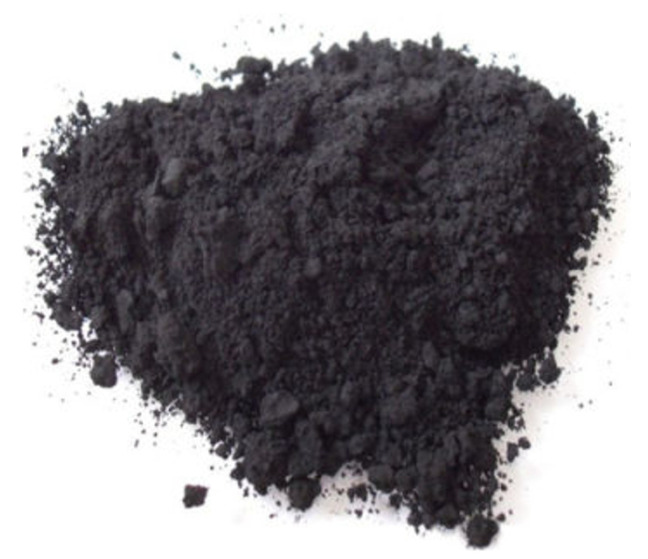
Appearance of MWCNT powder used in this study.

**Figure 2:**
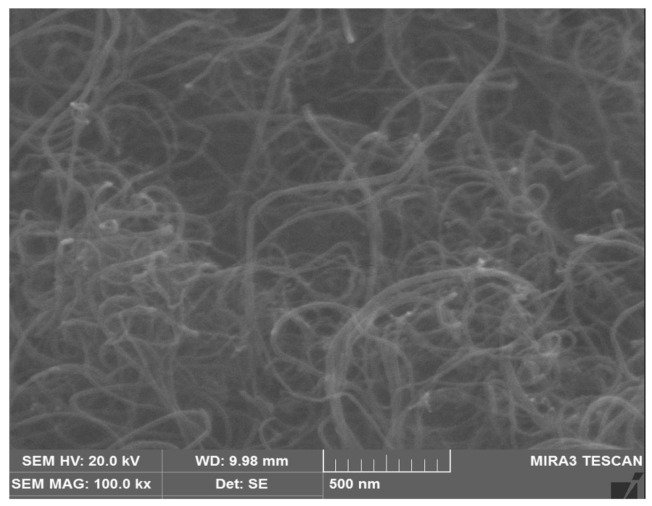
FE-SEM images of MWCNT powder (×100,000).

**Figure 3:**
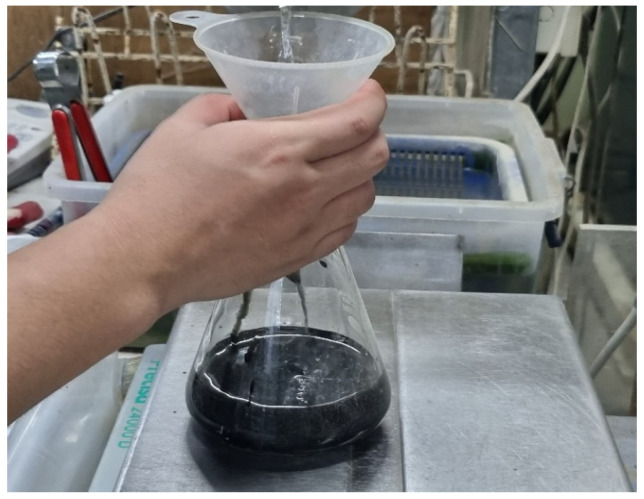
MWCNT dispersion.

The main properties of the MWCNT powder used in this study are shown in Table 3. Figure 1 shows the appearance of the MWCNT powder used. In addition, Figure 2 is an FE-SEM image of the MWCNT powder, taken at 100,000× magnification. The MWCNT powder used has the form of a bundle with a diameter of 10 to 100 nm, a length of 0.1 to 5 μm, a purity of 96.5 wt.% or higher, a bulk density of 0.08 to 0.12 g/cm^3^, and an electrical resistivity of 10^−3^ to 10^−6^ Ω·cm. When MWCNT powder is directly added during mixing, van der Waals forces are generated, causing entanglement and agglomeration between particles, making even dispersion difficult. The MWCNT dispersion used in this study was purchased in the form of a water-based suspension fabricated by U Company (Confluence, PA, USA), a specialized domestic manufacturer. The MWCNT dispersion was prepared at a concentration of 2.0 wt.% and was stably dispersed through the use of a chemical surfactant and ultrasonic treatment. Figure 3 shows the preparation of MWCNTs relatively evenly dispersed in an MWCNT aqueous solution.

### 2.2. Preparation of MWCNT Coating Solution

**Figure 4:**
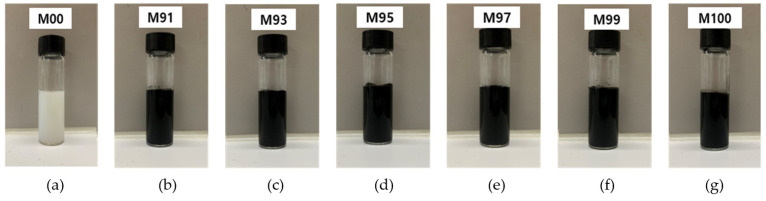
MWCNT-coated solutions prepared at various mixing ratios of MWCNT and PUD: (**a**) M00, (**b**) M91, (**c**) M93, (**d**) M95, (**e**) M97, (**f**) M99, and (**g**) M100.

To provide conductivity to the surface of the PET film, 2.0 wt.% of MWCNT was used, and for stable coating, a water-dispersed polyurethane solution (polyurethane dispersion, PUD, solid 40%, particle size < 200 nm, pH 8.0) from Company A (San Clemente, CA, USA) was used. A 2.0 wt.% MWCNT aqueous solution was added to a vial containing an aqueous polyurethane solution at a certain ratio and was mixed at 500 rpm for 10 min to prepare an MWCNT coating solution. Figure 4 is a photograph of a solution mixed with water-dispersed polyurethane at various mixing ratios of MWCNT: 0 wt.%, 91 wt.%, 93 wt.%, 95 wt.%, 97 wt.%, 99 wt.%, and 100 wt.%. The coating solution without MWCNT addition (M00) exhibited a white opaque color, whereas all coating solutions with MWCNT addition exhibited a black color. In particular, since the solution appears evenly black overall, it can be confirmed that the MWCNTs are evenly mixed with polyurethane and can be used as a solution for fabricating MWCNT-coated conductive films.

### 2.3. Preparation of Conductive Films Coated with MWCNT

**Figure 5:**
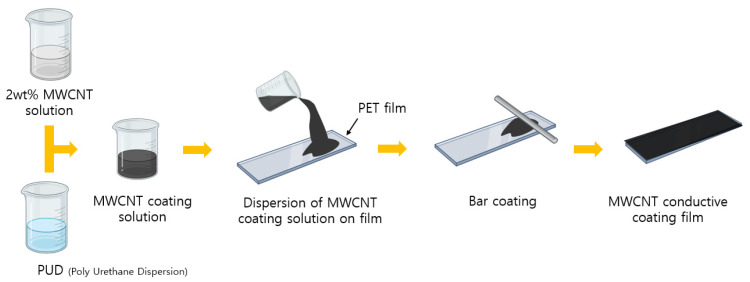
Schematic diagram of the preparation of MWCNT-coated conductive film.

The mixing ratio of the coating solution is shown in Table 4, and the process of fabricating conductive films coated with MWCNT by using the MWCNT coating solution is schematically shown in Figure 5. To proceed with the coating, the surface of a rectangular PET film (30 × 140 mm^2^) was washed with ethanol and dried. Then, to modify the surface to be hydrophilic, it was placed in the chamber of a plasma treatment machine (CUTE, Femto Science, Hwaseong-si, Republic of Korea), and a vacuum state of 5 × 10^−1^ Torr was created using a vacuum pump. Afterwards, surface treatment was performed for 10 min according to the basic settings of the equipment while injecting oxygen at a flow rate of 30 sccm. The plasma treatment was performed for 10 min at a power of 100 W under these conditions. These parameters were set according to the standard operation mode of the equipment to ensure uniform surface modification. Two mL of MWCNT coating solution was evenly applied to the surface of the surface-modified PET film using a bar coater (No. 24, RDS, Chelmsford, MA, USA), and then dried at room temperature for 24 h to form a coating film.

**Figure 6:**
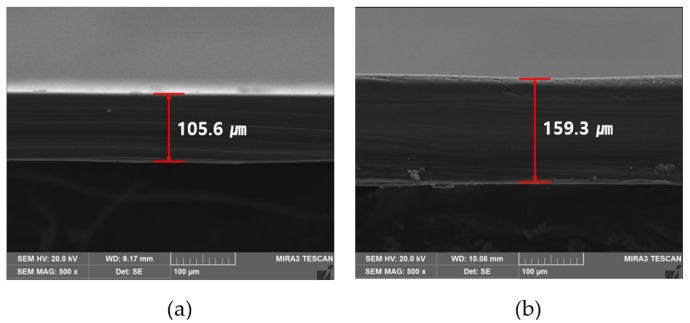
FE-SEM images of PET films: (**a**) uncoated transparent film (×500), and (**b**) MWCNT-coated film (×500).

Meanwhile, Figure 6 shows a photograph taken at 500× magnification of the PET film used in this study. Figure 6a shows a cross-section of an uncoated PET transparent film, and the thickness is approximately 105.6 μm. On the other hand, Figure 6b shows a cross-section of a coated PET film, and the total thickness was confirmed to be approximately 159.3 μm. The coating thickness was measured at three different locations along the film, with a deviation within ±0.3 μm, demonstrating a high degree of uniformity. This variation was determined as the maximum difference between an individual measurement and the average thickness (53.7 μm).

### 2.4. Experimental Paramerters

**Figure 7:**
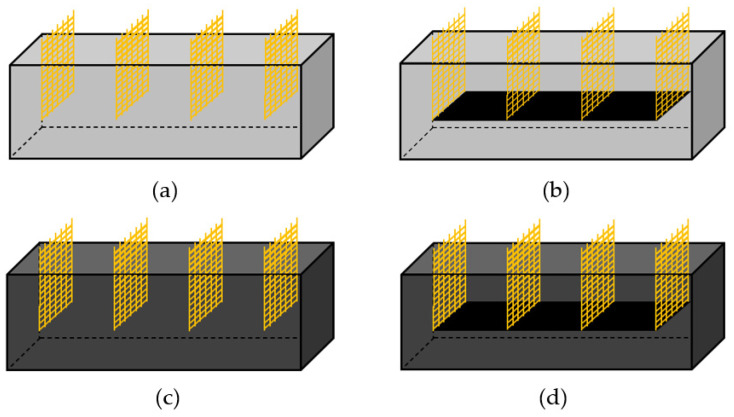
Schematic diagram of various specimens: (**a**) PCC, (**b**) MWCNT-coated film, (**c**) MWCNT dispersion, and (**d**) MWCNT-coated films and MWCNT dispersion.

The main experimental parameters and specimen names are listed in Table 5. Experimental parameters include the mixing method, MWCNT concentrations, use or absence of coating films, applied voltages, and electrode spacings. The mixing methods of the considered cement composites are divided into four groups. As shown in Figure 7, Group 1 is a specimen fabricated using PCC without either MWCNT dispersion or MWCNT-coated films. Group 2 is a specimen fabricated using only a film coated with an MWCNT in the PCC, while Group 3 is a specimen fabricated by directly mixing only MWCNT dispersion in the PCC. Finally, Group 4 is a specimen fabricated using both MWCNT dispersion and MWCNT-coated films in the PCC. Therefore, in this study, MWCNT dispersion was added at mixing ratios of 0.25 wt.%, 0.50 wt.%, and 0.75 wt.% relative to the cement weight, and all specimens were demolded 24 h after manufacture and then air-dried for 28 days. To analyze the heating performance, the applied direct current (DC) voltage was set to 10 V, 20 V, and 30 V, and the electrode spacing was 40 mm and 120 mm. However, when MWCNT is used at a high ratio, agglomeration occurs due to the strong van der Waals force between nanomaterials, which reduces dispersibility. Since this agglomeration phenomenon has a negative effect on the electrical and thermal properties of the coating films, M97 with an optimal mixing ratio of 97 wt.% or less was selected through experimental analysis in this study. In the case of Group 4 specimens, however, special care must be taken because there is a risk of the coating films becoming deformed or getting damaged when the electrode spacing is 40 mm and a voltage of 30 V or more is applied. In addition, according to KCS 14 20 10 [40] regulation, the temperature gradient is limited to 15 °C or less per hour, and the maximum temperature must be maintained at 65 °C or lower. This is because curing at a high temperature exceeding 65 °C may cause microcracks in the cement composites.

### 2.5. Mixing and Specimen Preparation

The mix proportions of cement composites are presented in Table 6 and are divided into four groups according to the mixing method. The water/cement ratio was maintained at 0.425, and the aggregate-to-cement ratio was selected as 1:2 by weight. It should be noted that the MWCNT concentration is proportional to the weight of cement. MWCNTs were added in proportions of 0.25 wt.%, 0.50 wt.%, and 0.75 wt.%, relative to the weight of cement, and the corresponding addition amounts are 0.2 g, 0.4 g, and 0.6 g, respectively. In addition, the cement composites using both MWCNT dispersion and MWCNT-coated films consist of two components in the total amount of MWCNT. That is, the amount of MWCNT dispersed in the aqueous solution and the amount of MWCNT included in the coating films are combined to form the total addition amount.

**Figure 8:**
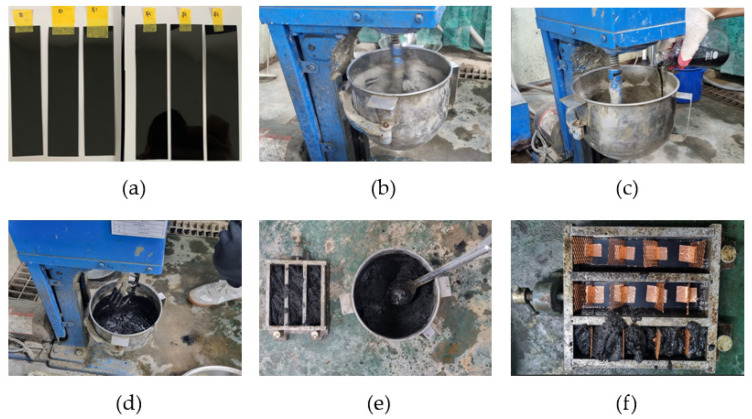
Specimen fabrication process: (**a**) prepared of MWCNT-coated films, (**b**) mixing of cement and sand in dry (120 s), (**c**) addition of MWCNT dispersion, (**d**) mixing of MWCNT dispersion (150 s), (**e**) pouring cement mixture into the mold, and (**f**) insertion of MWCNT-coated films.

Figure 8 illustrates the fabrication process of the conductive cement composite specimens. As described above, MWCNT dispersion was evenly coated on the entire surface of a transparent PET film using a bar coater method (Figure 8a). The amount of materials to be mixed is determined by the mixing ratio. The materials were mixed using a small Hobart-type cement mortar mixer with a capacity of approximately 4.73 L. To ensure uniform mixing, cement and fine aggregate were mixed at low speed for 30 s, and then dry mixed at high speed for 90 s (Figure 8b). After completing the dry mixing process, MWCNT dispersion and mixing water were added (Figure 8c) and mixed evenly for 90 s. Afterwards, the mixer was temporarily stopped, and the cement mixture attached to the bottom and walls of the container was removed using a rubber scraper, and then was mixed at high speed for 60 s (Figure 8d). After mixing, a certain amount of the cement mixture was taken out and slowly poured into the prepared mold (Figure 8e). For specimens using conductive coating films, the pre-prepared conductive coating films were carefully embedded after pouring the cement mixture (Figure 8f). After embedding the conductive coating films, the side of the mold was lightly tapped with a rubber mallet about 25 times to ensure that the cement mixture was sufficiently compacted and the layer was evenly formed. In the specimens using MWCNT-coated conductive films, copper C_u_ tape was firmly attached to the films using silver epoxy for electrical connection. This shall ensure that the electrical connection is maintained stably. Lastly, vibration compaction was performed on a vibrating table for approximately 2 min to minimize the voids in the specimens and ensure uniform contact with the copper mesh. All specimens were cured at room temperature for 24 h before being demolded, and then were left to dry naturally in the same environment for 27 days to minimize the influence of moisture. Meanwhile, the names of each specimen were determined as follows. First, the specimen without a dispersed MWCNT aqueous solution and conductive coating films was denoted as the PCC. On the other hand, the specimen using only MWCNT-coated film in the PCC was denoted as MCF, and the specimen using only MWCNT aqueous solution dispersed in the PCC was denoted as MD. Finally, the specimen using both MWCNT aqueous solution dispersed in the PCC and the conductive coating films was named MDCF. In addition, the second number included in each specimen name indicates the total concentration of MWCNT relative to the weight of cement, and is classified into three types: 0.25, 0.50, and 0.75 wt.%. For example, cement composites using a 0.25 wt.% MWCNT dispersion are denoted as MD-0.25.

**Figure 9:**
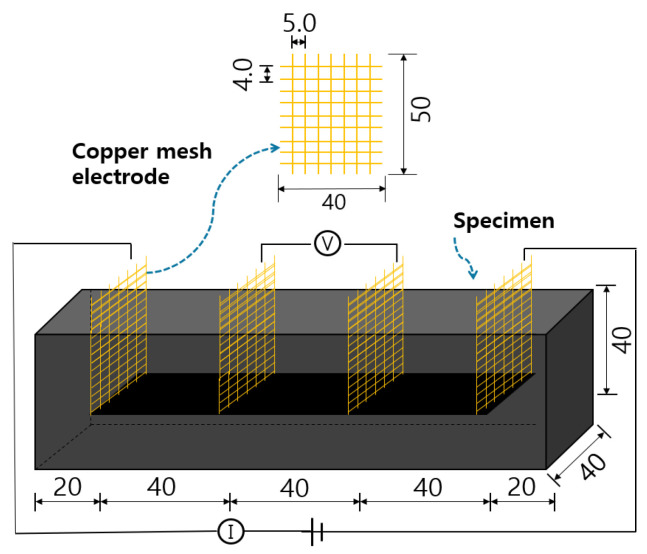
Four-electrode layout configuration for the heating performance and electrical resistance measurement of cement composites (Unit: mm).

In addition, as shown in Figure 9, the cement composites were embedded in a prism specimen measuring 40 × 40 × 160 mm^3^ in size with four copper mesh electrodes arranged at regular intervals. Each electrode was placed in a straight line at 40 mm intervals and embedded so that 20 mm protruded from the top of the specimen. Moreover, a copper mesh electrode was used at the contact point between the copper and cement composites to minimize discontinuity between different materials. Additionally, a copper mesh electrode was used at the contact point between the copper and cement composites to minimize discontinuity between different materials.

## 3. Test Methods

### 3.1. Transmittance Measurements of MWCNT-Coated Conductive Films

In this study, the transmittance of films coated with MWCNT at various mixing ratios was measured using a UV-visible spectrometer (UV-2450, Shimadzu, Kyoto, Japan) in the wavelength range of 200–800 nm.

### 3.2. FE-SEM Observations

FE-SEM (MIRA3-LMH, Tescan, Brno, Czechia) was employed to analyze the surface morphology of the coated conductive films and the distribution of MWCNT within the cement composites. For more precise analysis, the sample was thinly coated with platinum (Pt), and microstructures were photographed under an acceleration voltage of 10 kV. Before FE-SEM analysis, the cement composite specimens were crushed and dried in an electric oven at 65 °C for 1 day to remove free moisture.

### 3.3. Heating Performance Tests

**Figure 10:**
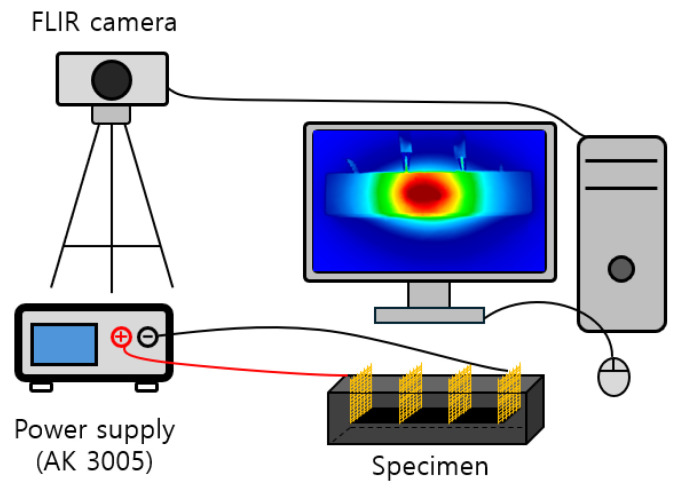
Experimental set-up of heating performance tests.

There are no separate domestic or foreign regulations for testing the heating performance and electrical resistance of cement composites. In this study, therefore, specimens measuring 40 × 40 × 160 mm^3^ were fabricated based on the domestic standard KS L ISO 679 [39] and the international standard ASTM C348 [41]. Figure 10 features the experimental setup for measuring the heating performance of cement composite specimens. The heating performance experiment was conducted by supplying a constant DC voltage to the specimen. In the experiment, clamps were connected to copper mesh electrodes spaced at 40 mm and 120 mm intervals, and various applied voltages (10 V, 20 V, 30 V) were supplied using a power supply (DC power supply, AK3005, Vupower, Yongin, Republic of Korea). Thereafter, the surface temperature was measured at 1-s intervals for 1 h (3600 s) using a thermal imaging camera (Infrared thermal camera, T630sc, FLIR, Wilsonville, OR, USA) while maintaining the initial temperature at 20 ± 0.5 °C. At the same time, the strength of the applied voltages was measured using a digital multimeter, and it was confirmed that a constant voltage was stably supplied. To prevent electric shock and short circuits and ensure safety during the experiment, voltage was supplied while all specimens were placed on the insulated rubber plates.

### 3.4. Measurements of Electrical Resistance

**Figure 11:**
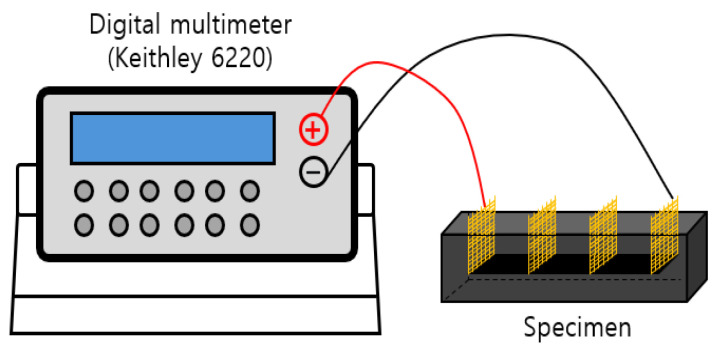
Experimental set-up of electrical resistance measurement.

Two-probe and four-probe methods are commonly used to measure the electrical resistance of cement composites. Although the two-probe circuit is simpler, the four-probe method is preferred because the contact resistance between the specimen and the electrodes can affect the measurements [42,43]. Figure 11 shows the experimental setup for measuring the electrical resistance of the cement composite specimens. In the present study, the electrical resistance of cement composites was measured using a digital multimeter (Keithley 6220, Keithley Instruments from Solon, Ohio, USA). The four-probe method was used for the measurement, and the electrodes of the films in the cement composites were connected using four probes, and then a voltage was applied. Afterwards, the electrical resistance value was measured according to the current and voltage changes flowing through the films. In order to obtain reliable electrical resistance data, each experimental variable was measured twice, and the average value was used to gain the data.

## 4. Results and Discussion

### 4.1. Transmittance and Surface Properties of MWCNT-Coated Conductive Films

**Figure 12:**
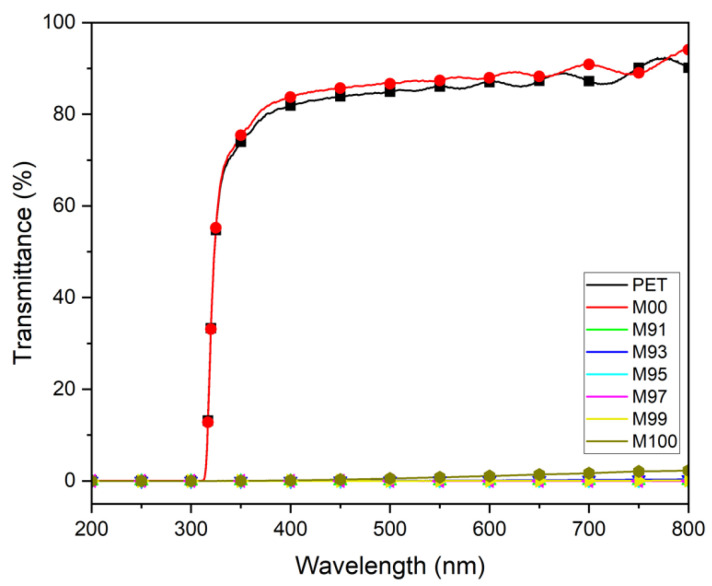
Transmittance of MWCNT-coated conductive films prepared at various composition ratios.

Figure 12 shows the transmittance of MWCNT-coated conductive films prepared with MWCNT at various mixing ratios. While the uncoated PET film and the M00 sample coated only with urethane showed high transmittance of about 86% in the visible light range, all MWCNT-coated conductive films showed transmittance of 0% in the visible light range. This indicates that MWCNTs were evenly formed as an MWCNT coating layer on the surface of the PET film.

**Figure 13:**
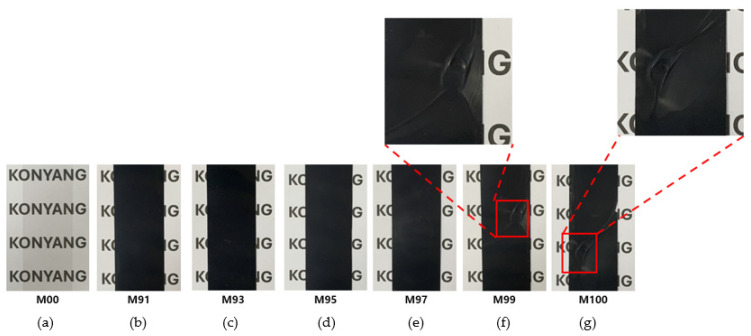
MWCNT-coated conductive films prepared at various composition ratios: (**a**) M00, (**b**) M91, (**c**) M93, (**d**) M95, (**e**) M97, (**f**) M99, and (**g**) M100.

Figure 13 is a photograph showing the surface morphology of MWCNT-coated conductive film prepared using coating solutions at various MWCNT composition ratios. Here, we could confirm that the coating film (M00) without MWCNT addition was transparent. On the other hand, the coating film with MWCNT addition all exhibited a black color. This means that MWCNTs were evenly coated on the entire surface of the PET film. However, since it was confirmed that MWCNT agglomerated partially in the coating film of the M99 and M100 samples, it is judged appropriate to adjust the mixing ratio of MWCNT to 97 wt.% or less. Therefore, when MWCNTs are used at a high ratio, agglomeration occurs due to the strong van der Waals force between nanomaterials, which reduces dispersibility. This agglomeration phenomenon can have a negative effect on the electrical and thermal properties of the coating films. Therefore, M97 coating film with the MWCNT incorporation ratio of 97 wt.% was utilized in the manufacture of the exothermic cement composites.

**Figure 14:**
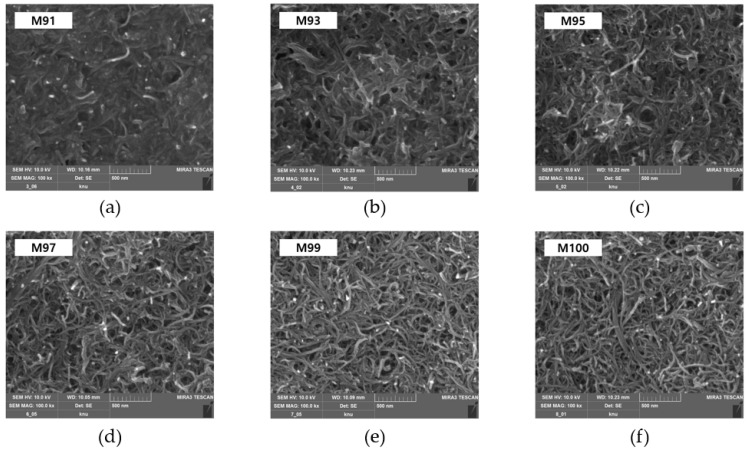
FE-SEM images of MWCNT-coated conductive films prepared at various composition ratios: (**a**) M91, (**b**) M93, (**c**) M95, (**d**) M97, (**e**) M99, and (**f**) M100.

Figure 14, captured by FE-SEM, displays the surface morphology of MWCNT-coated conductive films fabricated at various mixing ratios. Here, we can confirm that MWCNTs with a thickness of 20 to 30 nm are evenly dispersed on the surface of the conductive coating films, forming a uniform coating layer. When the mixing ratio of MWCNT is low, urethane fills between the MWCNT, hindering the formation of a conductive network, but as the mixing ratio increases, it can be seen that a network is formed directly or indirectly between the MWCNT.

### 4.2. Heating Performance and Thermal Image Analysis

Table 7 summarizes the experimental results of measuring the heating performance of cement composites fabricated at various mixing ratios, applied voltages, and different electrode spacings. The surface temperature of the specimen was measured by adding the heat from the applied voltage to the initial temperature (approximately 20.0 ± 0.5 °C). In this experiment, after applying various voltages for 1 h, the heating performance with respect to electrode spacing was compared and analyzed. For the PCC specimen without either MWCNT dispersion or MWCNT-coated films, the temperature increase was only 0.2 °C at the maximum, and the heating performance was significantly lower at 76.9 °C compared to the MDCF-0.75 specimen. The highest calorific value was measured at 97.1 °C in the MDCF-0.75 specimen with an electrode gap of 40 mm and an applied voltage of 30 V. This might be analyzed as a result of the formation of a denser and more continuous MWCNT network since MWCNT is evenly dispersed throughout the cement matrix via dispersion and the coating films. As the MWCNT concentration increased, the heating performance tended to improve, and in particular, as the electrode spacing narrowed, the electrical resistance decreased, which further improved the heating performance. This is because the narrower the electrode spacing is, the smoother the current flow becomes, and the less electrical resistance loss there is, the greater the heating effect.

**Figure 15:**
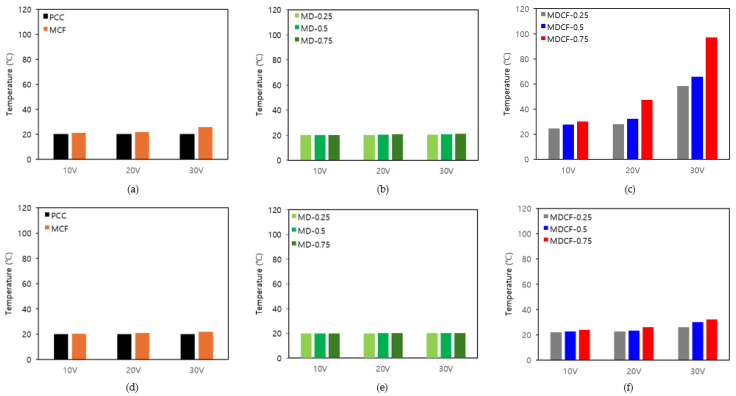
Variations in peak temperature under various applied voltages and different electrode spacings: (**a**) specimens using PCC and only MWCNT-coated films (electrodes spacing 40 mm), (**b**) specimens using PCC and only MWCNT dispersion (electrodes spacing 40 mm), (**c**) specimens using both MWCNT-coated films and MWCNT dispersion (electrodes spacing 40 mm), (**d**) specimens using PCC and only MWCNT-coated films (electrodes spacing 120 mm), (**e**) specimens using PCC and only MWCNT dispersion (electrodes spacing 120 mm), and (**f**) specimens using both MWCNT-coated films and MWCNT dispersion (electrodes spacing 120 mm).

Figure 15 is a graph showing the maximum temperature measurement results of cement composites according to various applied voltages and different electrode spacings. Figure 15a displays the results of comparing the maximum temperatures of the PCC specimen without either MWCNT dispersion or MWCNT-coated film, and the MCF specimen with only MWCNT-coated films at various applied voltages and an electrode spacing of 40 mm. While the PCC specimen showed almost no temperature increase as the applied voltage increased, the MCF specimen showed a slightly higher heating effect, with a temperature increase of approximately 5.8 °C at an applied voltage of 30 V. Figure 15b shows the results comparing the maximum temperatures of the MD specimens using only MWCNT dispersion at various applied voltages and an electrode spacing of 40 mm. When the applied voltage was 30 V, the MD-0.25 specimen showed a temperature increase of 0.4 °C, the MD-0.50 specimen showed a temperature increase of 0.7 °C, and the MD-0.75 specimen showed a temperature increase of 1.0 °C. Even when the applied voltage was 30 V, the heating performance of the cement composites was not significantly improved. This indicates that it is difficult to effectively improve the self-heating properties of the cement composites using only MWCNT dispersion. Figure 15c displays the results of comparing the maximum temperatures of MDCF specimens using both MWCNT dispersion and MWCNT-coated films at various applied voltages, with an electrode spacing of 40 mm. When the applied voltage was 30 V, the MDCF-0.25 specimen showed a temperature increase of 38.5 °C, the MDCF-0.50 specimen featured a temperature increase of 45.7 °C, and the MDCF-0.75 specimen indicated a temperature increase of 77.1 °C. At the same applied voltage of 30 V, particularly, the MDCF-0.75 specimen using both the 0.75 wt.% MWCNT dispersion and MWCNT-coated film showed a temperature increase that was approximately 77.1 times higher than that of the MD-0.75 specimen using only MWCNT dispersion at the same concentration, and approximately 13.3 times higher than that of the MCF specimen using only MWCNT-coated film. This shows that the heating performance can be further improved when both MWCNT dispersion and MWCNT-coated films are used, and it is analyzed that the conductive coating films facilitate the flow of current, thereby generating heat more efficiently. Using both, the heating performance becomes more uniform and optimized, and it tends to maintain a temperature above a certain level even at a low voltage of 20 V, suggesting its potential as an energy-efficient heating material. Figure 15d indicates the results of comparing the maximum temperatures of the PCC specimen without either MWCNT dispersion or MWCNT-coated film, and the MCF specimen with only MWCNT-coated films at various applied voltages and an electrode spacing of 120 mm. In all specimens, the temperature increase was minimal despite the various changes in applied voltage, confirming that there was no significant difference in heating performance. Figure 15e shows the results comparing the maximum temperatures of the MD specimens using only MWCNT dispersion at various applied voltages and an electrode spacing of 120 mm. When the applied voltage was 30 V, the temperature increase was 0.1 °C for the MD-0.25 specimen, 0.2 °C for the MD-0.50 specimen, and 0.3 °C for the MD-0.75 specimen, indicating that there was almost no temperature increase. This means that the specimen with an electrode spacing of 120 mm had relatively lower heating performance than the specimen with an electrode spacing of 40 mm. Figure 15f displays the results of comparing the maximum temperatures of MDCF specimens using both MWCNT dispersion and MWCNT-coated films at various applied voltages and an electrode spacing of 120 mm. When the applied voltage was 30 V, the temperature increase was 6.1 °C in the MDCF-0.25 specimen, 10.1 °C in the MDCF-0.50 specimen, and 12.3 °C in the MDCF-0.75 specimen, respectively. Moreover, the heating performance was relatively low in the Group 4 specimens with an electrode spacing of 120 mm. When comparing the temperature increase according to different electrode spacings, the MDCF-0.25 specimen turned out to be 32.4 °C, the MDCF-0.50 specimen showed 35.6 °C, and the MDCF-0.75 specimen featured the highest temperature increase at 64.8 °C. This may be analyzed as being because the closer the electrode spacing, the more smoothly the current flows through the shortest distance, reducing the electrical resistance, and as a result, the temperature of the cement composites increases further.

**Figure 16:**
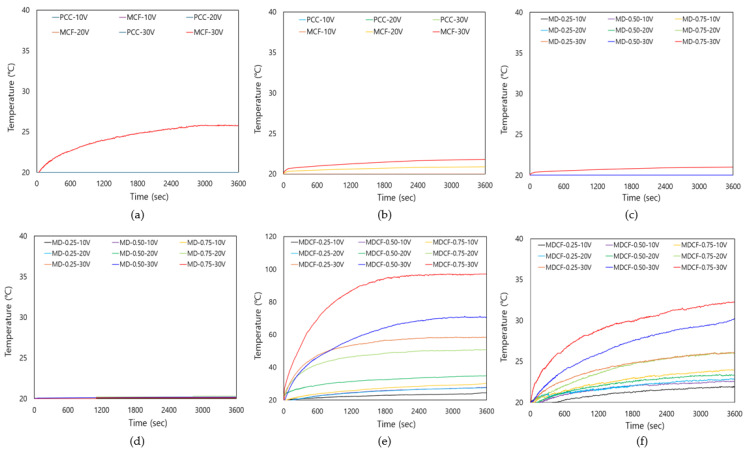
Temperature-elapsed time curves: (**a**) specimens using PCC and only MWCNT-coated films (electrodes spacing 40 mm), (**b**) specimens using PCC and only MWCNT-coated films (electrodes spacing 120 mm), (**c**) specimens using PCC and only MWCNT dispersion (electrodes spacing 40 mm), (**d**) specimens using PCC and only MWCNT dispersion (electrodes spacing 120 mm), (**e**) specimens using both MWCNT-coated films and MWCNT dispersion (electrodes spacing 40 mm), and (**f**) specimens using both MWCNT-coated films and MWCNT dispersion (electrodes spacing 120 mm).

Figure 16 is a graph revealing the change in surface temperature over time of the cement composites. Figure 16a,b show the change in surface temperature of the PCC specimen and the MCF specimen using only MWCNT-coated film in the PCC under various applied voltages and different electrode spacings. In the case of the PCC specimen, the temperature increase was not large even when the applied voltage increased, and overall, a minimal temperature increase was observed. This is analyzed as being due to the low conductivity of the PCC itself. In addition, the heating performance was not significantly improved in the MCF specimen using only MWCNT-coated film, and a similar level of temperature increase was observed in both cases where the electrode spacing was 40 mm and 120 mm. This seems to be because there is a limit to forming a sufficient conductive network using only MWCNT-coated films. Figure 16c,d show the surface temperature changes of the MD specimen that uses only MWCNT dispersion in the PCC. Even in this case, the difference in heating performance when the electrode spacing was 40 mm and 120 mm was not significant, and the temperature increase tended to remain low over time. This suggests that it is difficult to sufficiently secure heating performance by using only MWCNT dispersion. In addition, even when the MWCNT concentration increased, there was no significant difference in the temperature gradient, and it tended to maintain a constant temperature after reaching the peak. Figure 16e,f reveal the surface temperature changes of the MDCF specimen using both MWCNT dispersion and MWCNT-coated films in the PCC. The temperature gradient and final temperature varied depending on the mixing method used. The temperature gradient was relatively high compared to that of specimens using only MWCNT dispersion or only MWCNT-coated films. This is analyzed as a result of MWCNT being evenly dispersed in an aqueous solution in the PCC, forming a conductive network with the coating films, thereby improving thermal conductivity and increasing temperature and heat diffusion. It increased as the MWCNT concentration increased, and the temperature stabilized when it reached the maximum temperature. In particular, when the electrode spacing was 40 mm, the temperature gradient was steeper than when the electrode spacing was 120 mm. This is analyzed to be because the narrower the electrode spacing, the smoother the current flows within the composite, leading to a relatively higher temperature increase. Therefore, the analysis results found that the heating performance of the PCC itself is very low, and the heating effect is not significant even when only coating films or dispersion of MWCNT is used. However, it was confirmed that when both MWCNT-coated films and dispersion of MWCNT were used, the heating performance was significantly improved, and the temperature increase tended to be higher as the electrode gap narrowed. It seems thus necessary to use both MWCNT dispersion and MWCNT-coated films in order to secure effective heating performance, and the optimization of the electrode gap is also considered an important factor.

**Figure 17:**
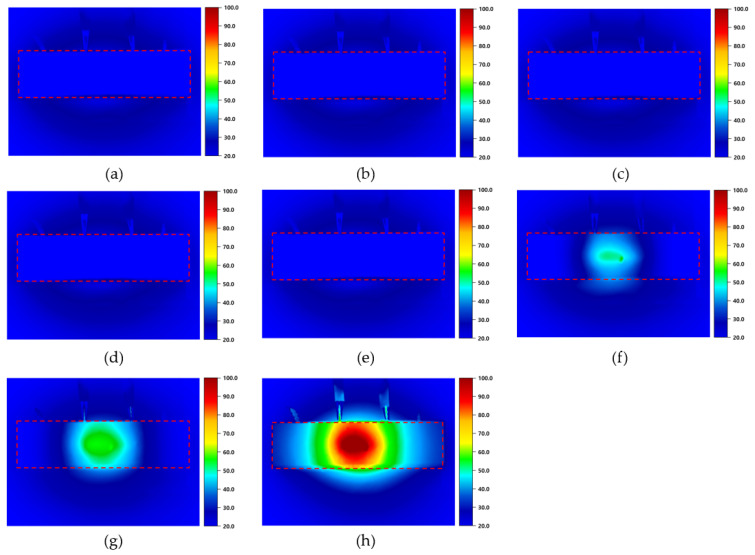
Thermal images of specimens with 40 mm spacing between electrodes under an applied voltage of 30 V: (**a**) PCC (20.2 °C), (**b**) MCF (25.8 °C), (**c**) MD-0.25 (20.4 °C), (**d**) MD-0.50 (20.7 °C), (**e**) MD-0.75 (21.0 °C), (**f**) MDCF-0.25 (58.5 °C), (**g**) MDCF-0.50 (65.7 °C), and (**h**) MDCF-0.75 (97.1 °C). Red dashed boxes highlight the regions of interest where temperature measurements were focused.

Figure 17 shows a thermal image taken when the maximum surface temperature of the cement composites was reached, as the electrode spacing was 40 mm and the applied voltage was 30 V. This allows for visual confirmation of the heating performance and surface temperature distribution of the specimen. For example, the surface temperature is calculated by adding the temperature increase caused by the applied voltage to the initial temperature. This means that, if the initial temperature of the MDCF-0.75 specimen is 20.0 °C and the temperature increases to 77.1 °C due to the applied voltage, the final surface temperature can be calculated as 97.1 °C. In Figure 17a,b, we can see thermal images of the PCC specimen without either MWCNT dispersion or MWCNT-coated film, and the MCF specimen with only MWCNT-coated film. The surface temperatures of the PCC and MCF specimens were measured to be 20.2 °C and 25.8 °C, respectively. Since the temperature increase was relatively small, the surface temperature of the specimen showed little difference from the surrounding environment in the thermal image. Figure 17c–e are thermal images of the MD specimens using only MWCNT dispersion. The surface temperatures of the MD-0.25, MD-0.50, and MD-0.75 specimens were measured to be 20.4 °C, 20.7 °C, and 21.0 °C, respectively. Since, however, the temperature increase was very small, the change in the surface temperature of the specimen was not large, and it was thus difficult to confirm a clear temperature distribution even in the thermal image. Figure 17f–h feature thermal images of the MDCF specimen using both MWCNT dispersion and MWCNT-coated films. The surface temperatures of the MDCF-0.25, MDCF-0.50, and MDCF-0.75 specimens were measured as 58.5 °C, 65.7 °C, and 97.1 °C, respectively. Meanwhile, the MDF-0.75 specimen showed the highest surface temperature. MDCF specimens using both MWCNT dispersion and MWCNT-coated films exhibited higher temperatures than MD specimens using only MWCNT dispersion or MCF specimens using only MWCNT-coated films. Particularly, such a trend was confirmed in all specimens with MWCNT concentrations of 0.25 wt.%, 0.50 wt.%, and 0.75 wt.% at an electrode spacing of 40 mm and an applied voltage of 30 V. This is because the MWCNTs were evenly dispersed in the films, forming a continuous MWCNT network throughout the specimens. These networks can serve to enhance the electrical conductivity and thus further increase the heating performance of the cement composites. Furthermore, at the same applied voltage, the surface temperature increased as the MWCNT concentration increased, and a clearer thermal image was formed. This is because heat is mainly generated between the electrodes connected to the power supply, resulting in the current traveling along the shortest path. It is observed that the resulting heat is mainly concentrated and distributed between the electrodes.

### 4.3. Electrical Properties

Table 8 summarizes the results of electrical resistance measurements according to different electrode spacings of cement composites fabricated at various mixing ratios. The electrical resistance values of the PCC specimen without either MWCNT dispersion or MWCNT-coated films, and of the MCF specimen with only MWCNT-coated films, were measured to be 88,110 Ω and 75,140 Ω, respectively, at an electrode spacing of 40 mm. On the other hand, the electrical resistance value of the MDCF-0.75 specimen using both MWCNT dispersion and MWCNT-coated films was 180 Ω, which was the lowest value among all specimens. These values are approximately 489 times and 417 times higher than those of the PCC and MCF specimens, respectively, indicating that the conductivity is significantly improved when both MWCNT dispersion and MWCNT-coated films are used. This suggests that the uniform dispersion of MWCNT and the effective enhancement of the conductivity of the coating films could contribute to the reduction of electrical resistance. In particular, this is believed to be because the electrical resistance showed a tendency to gradually decrease as the MWCNT concentration increased, and the MWCNT network became denser with increasing MWCNT concentration. Moreover, as the electrode spacing becomes narrower, the electric resistance tends to decrease somewhat because the current flows along the shortest path within the cement matrix. However, the difference in electric resistance with respect to the electrode spacing was confirmed to be relatively small.

**Figure 18:**
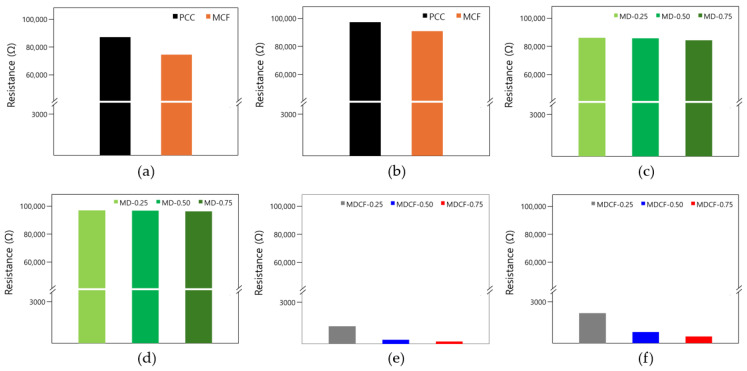
Electrical resistance with various experimental parameters: (**a**) specimens using PCC and only MWCNT-coated films (electrodes spacing 40 mm), (**b**) specimens using PCC and only MWCNT-coated films (electrodes spacing 120 mm), (**c**) specimens using PCC and only MWCNT dispersion (electrodes spacing 40 mm), (**d**) specimens using PCC and only MWCNT dispersion (electrodes spacing 120 mm), (**e**) specimens using both MWCNT-coated films and MWCNT dispersion (electrodes spacing 40 mm), and (**f**) specimens using both MWCNT-coated films and MWCNT dispersion (electrodes spacing 120 mm).

Figure 18 is a graph showing the change in electrical resistance according to various experimental parameters of the cement composites. Figure 18a shows the change in electrical resistance of the PCC specimen without either MWCNT dispersion or MWCNT-coated film, and that of the MCF specimen with only MWCNT-coated films when the electrode spacing is 40 mm. The electrical resistance values of the PCC and MCF specimens were measured as 88,110 Ω and 75,140 Ω, respectively. All specimens showed high electrical resistance, indicating that they had little electrical performance. Even when only MWCNT-coated films were used, the formation of a conductive network was limited, and the electrical properties were not significantly improved despite the use of the coating films. Figure 18b shows the change in electrical resistance of the PCC specimen without either MWCNT dispersion or MWCNT-coated film, and of the MCF specimen with only MWCNT-coated film when the electrode spacing is 120 mm. The electrical resistance values of the PCC and MCF specimens were measured as 98,590 Ω and 91,980 Ω, respectively. In particular, the electrical resistance was measured to be the highest for the PCC specimen, which is analyzed to be the result of the greatest increase in electrical resistance due to the lack of conductive materials. Figure 18c shows the change in electrical resistance of the MD specimen that uses only MWCNT dispersion when the electrode spacing is 40 mm. The electrical resistance values of the MD-0.25, MD-0.50, and MD-0.75 specimens were measured as 87,030 Ω, 86,760 Ω, and 85,250 Ω, respectively. As the MWCNT concentration increased, the electrical resistance showed a gradual tendency to decrease slightly, but the difference was not large, and the conductivity did not significantly improve. Figure 18d shows the change in electrical resistance of the MD specimen that uses only MWCNT dispersion when the electrode spacing is 120 mm. The electrical resistance values of the MD-0.25, MD-0.50, and MD-0.75 specimens were measured as 98,260 Ω, 98,040 Ω, and 97,520 Ω, respectively. Likewise, all specimens still showed high electrical resistance, indicating little electrical properties, which means that MWCNT did not sufficiently form a conductive network within the cement composites. Figure 18e shows the change in electrical resistance of the MDCF specimen using both MWCNT dispersion and MWCNT-coated films when the electrode spacing is 40 mm. The electrical resistance values of the MDCF-0.25, MDCF-0.50, and MDCF-0.75 specimens were measured to be 1300 Ω, 320 Ω, and 180 Ω, respectively. By employing both the dispersion and the coating films of MWCNT, a conductive network can be effectively formed within the cement matrix, and can significantly reduce the electrical resistance. In addition, the fact that the difference in electrical resistance of each specimen is not large suggests that the MWCNTs were relatively evenly dispersed within the cement matrix and that a continuous path for electron transfer was effectively formed. Thus, it can be confirmed that using both MWCNT dispersion and MWCNT-coated films is very effective in improving the electrical performance. Figure 18f indicates the change in electrical resistance of the MDCF specimen using both MWCNT dispersion and MWCNT-coated films when the electrode spacing is 120 mm. The electrical resistance values of the MDCF-0.25, MDCF-0.50, and MDCF-0.75 specimens were measured as 2220 Ω, 820 Ω, and 590 Ω, respectively. These electrical resistance values were 1.7, 2.5, and 3.3 times higher, respectively, than when the electrode spacing was 40 mm. This is considered to be due to the fact that the overall electrical resistance increased as the current flow path became longer with the increase in electrode spacing.

### 4.4. FE-SEM Image Analysis

**Figure 19:**
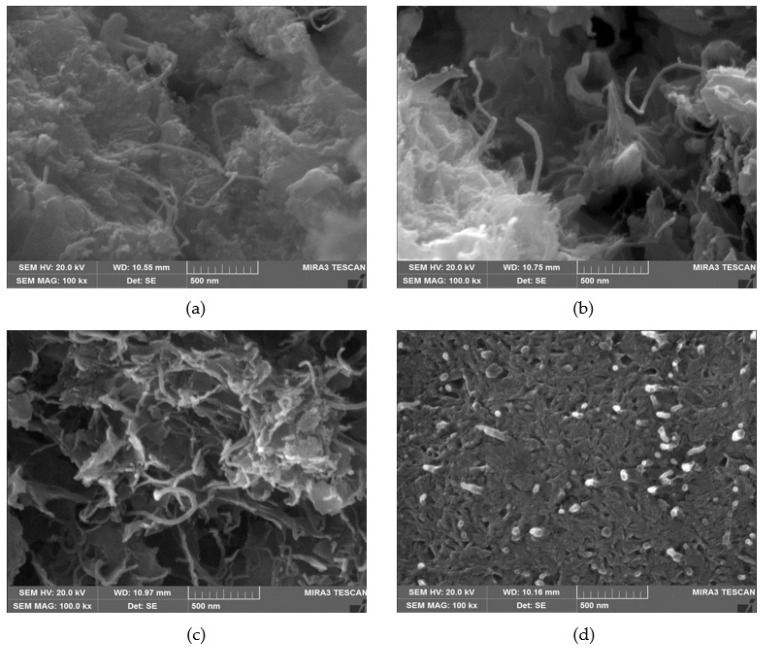
FE-SEM images of cement composites: (**a**) 0.25 wt.% MWCNT dispersion (×100,000), (**b**) 0.50 wt.% MWCNT dispersion (×100,000), (**c**) 0.75 wt.% MWCNT dispersion (×100,000), and (**d**) 0.75 wt.% MWCNT dispersion and MWCNT-coated film (×100,000).

The interfacial transition zone (ITZ) between the matrix and aggregate in cement composites generally has a thickness of 10 to 50 μm, which has a significant impact on the strength and durability of cement composites. It has been reported that the properties and formation process of ITZ serve as key factors in material performance, and the formation of a conductive network in this region plays an important role in improving the electrical and heating performance of the composite [44,45]. Figure 19 is a FE-SEM image showing the microstructure of cement composites using various mixing ratios and conductive coating films. Figure 19a shows the microstructure image of the cement composites using only 0.25 wt.% MWCNT dispersion. As can be seen in the image, MWCNTs were not uniformly distributed within the cement composites, and, in some areas, they are not sufficiently spread, indicating limited network formation. In addition, a large number of pores were generated around the aggregates within the cement composites, which tended to deteriorate the homogeneity of the matrix. Particularly, since the MWCNT failed to form a continuous network within the matrix, the electrical connectivity was poor, which resulted in a decrease in the heating performance. Figure 19b reveals the microstructural image of the cement composites using only 0.50 wt.% MWCNT dispersion. Compared to 0.25 wt.%, MWCNTs were distributed more uniformly, and a continuous network tended to form in some areas. However, it was still difficult to form a complete network structure due to the insufficient connectivity among MWCNTs. Such a phenomenon is thought to be because MWCNTs have a low affinity for cement particles and hydration products, making it difficult to bind, and their interfacial bonding with the matrix is weak. Figure 19c reveals the microstructural image of the cement composites using only 0.75 wt.% MWCNT dispersion. Compared to 0.25 wt.% and 0.50 wt.%, the distribution of MWCNT looked somewhat more uniform, but in some areas, an uneven distribution still appeared, indicating that network formation was not sufficient. Furthermore, although conductivity is expected to improve as the MWCNT concentration increases, it was difficult to form a continuous network because sufficient connectivity between the MWCNTs was not ensured. Such distribution properties might cause deterioration of the heating performance and an increase in the electrical resistance of the composite. Figure 19d shows the microstructural image of the cement composites using both the 0.75 wt.% MWCNT dispersion and MWCNT-coated films. A large amount of MWCNTs were observed on the surface of the coating films, indicating that the MWCNTs were evenly distributed, which improved electrical connectivity and enhanced heating performance and conductivity. Moreover, the cement composites using both the 0.75 wt.% MWCNT dispersion and MWCNT-coated films formed a more dense and uniform internal structure, which suggests that this might contribute to the improvement of the heating performance and electrical properties. In sum, the FE-SEM analysis results confirmed that the MWCNT network was formed on the surface of the MWCNT coating films, which enhanced the electrical connection and thereby improved the heating performance and reduced the electrical resistance. This is consistent with the results reported in some previous studies, which showed that when a continuous MWCNT network structure is formed, electron movement becomes smoother and conductivity increases [46,47].

## 5. Conclusions

In this study, conductive films coated with MWCNT were fabricated, and their effects on the heating performance and electrical properties of cement composites were analyzed. The heating performance and electrical properties of cement composites were characterized, and the main results obtained through this study are as follows.

1. MWCNT-coated conductive films were fabricated by evenly applying MWCNT to the entire front surface of a PET film substrate using a bar-coating method. As a result, it was confirmed that when the mixing ratio of MWCNT was 99% or higher, agglomeration occurred on the film surface, whereas when it was 97% or lower, a uniform coating was formed. This serves as evidence that a mixing ratio of 97% or lower is optimal.
2. When the electrode spacing was 40 mm and the applied voltage was 30 V, the MDCF-0.75 specimen using both MWCNT dispersion and MWCNT-coated films showed the highest heating performance, with the temperature increasing up to a maximum of 77.1 °C. At the same time, this specimen showed the lowest electrical resistance of 180 Ω. This is analyzed as a result of MWCNT being evenly dispersed not only in the coated films but also within the cement composites, forming a denser MWCNT network. It was thus possible to confirm that using both MWCNT dispersion and MWCNT-coated films is the optimal method to effectively improve the heating performance and electrical conductivity of cement composites.
3. As the concentration of MWCNT increased, the heating performance improved and the electrical resistance decreased. This is analyzed as a result of the MWCNT networks forming more densely within the cement composites as the concentration of conductive MWCNT increased.
4. As the electrode spacing became narrower, the heating performance increased significantly and the electrical resistance decreased further. This is because as the electrode spacing became narrower, the current flow became smoother and the loss due to electrical resistance decreased, further increasing the heating effect.
5. FE-SEM analysis results showed that the network formation of MWCNT-coated films enhanced the electrical connection, thereby improving the heating performance and reducing the electrical resistance. In particular, the cement composites using both 0.75 wt.% MWCNT dispersion and MWCNT-coated films formed a denser and more uniform internal structure, suggesting that the electrical properties and heating performance can be effectively improved.

## Figures and Tables

**Table 1:** Physical properties of standard sand.

Size (mm)	Unit Weight (kg/m^3^)	Density (g/cm^3^)	Percentage Water Absorption (%)	Fineness Modulus (FM)
≤2	1490	2.65	0.1	2.40

**Table 2:** Main properties of PET.

Thickness (μm)	Heat Shrinkage (%)	Tensile Strength (MPa)	Elongation at Break (%)	Surface Tension (mN/m)
100 ± 2.5	≤0.5~1.5	205 ± 49	160 ± 50	54 ± 3

**Table 3:** Main properties of MWCNT powder.

Diameter (nm)	Length (μm)	Purity (wt.%)	Specific Surface Area (m^2^/g)	Bulk Density (g/cm^3^)	Resistivity (Ω·cm)	Thermal Conductivity (W/m·K)
10~100	0.1~5	>96.5	200~250	0.08~0.12	10^−3^~10^−6^	Max 3000

**Table 4:** Composition of the MWCNT coating solutions prepared at various mixing ratios of MWCNT and PUD.

Sample Name	Mixing Ratios (wt.%)	
	MWCNT	PUD
M00	00	100
M91	91	9
M93	93	7
M95	95	5
M97	97	3
M99	99	1
M100	100	0

**Table 5:** Experimental parameters and specimen designations.

Mixing Method	Specimen ID	MWCNT Concentration (wt.%)	Use of MWCNT-Coated Films	Electrodes Spacing (mm)	Applied Voltage (V)
Plain cement composites (Group 1)	PCC	0.00	None	40/120	10/20/30
MWCNT-coated film (Group 2)	MCF	0.00	Used	40/120	10/20/30
MWCNT dispersion (Group 3)	MD-0.25	0.25	None	40/120	10/20/30
	MD-0.50	0.50	None		
	MD-0.75	0.75	None		
MWCNT-coated films and MWCNT dispersion (Group 4)	MDCF-0.25	0.25	Used	40/120	10/20/30
	MDCF-0.50	0.50	Used		
	MDCF-0.75	0.75	Used		

**Table 6:** Mix proportions of cement composites.

Specimen ID	Water/Cement (%)	Cement/Fine Aggregate	MWCNT Concentration (wt.%)	Use of MWCNT-Coated Films	Cement (g)	Fine Aggregate (g)	Water (g)
PCC	42.5	1:2	0.00	None	80	160	34
MCF	42.5	1:2	0.00	Used	80	160	34
MD-0.25	42.5	1:2	0.25	None	80	160	34
MD-0.50			0.50	None			
MD-0.75			0.75	None			
MDCF-0.25	42.5	1:2	0.25	Used	80	160	34
MDCF-0.50			0.50	Used			
MDCF-0.75			0.75	Used			

**Table 7:** Results of heating performance tests.

Specimen ID	MWCNT Concentration (wt.%)	Use of MWCNT-Coated Films	Maximum Heating Performance (°C)					
			At 40 mm Spacing			At 120 mm Spacing		
			10 V	20 V	30 V	10 V	20 V	30 V
PCC	0.00	None	20.0	20.1	20.2	20.0	20.0	20.0
MCF	0.00	Used	21.2	21.9	25.8	20.3	20.9	21.8
MD-0.25	0.25	None	20.0	20.2	20.4	20.0	20.0	20.1
MD-0.50	0.50	None	20.1	20.4	20.7	20.0	20.1	20.2
MD-0.75	0.75	None	20.2	20.6	21.0	20.0	20.2	20.3
MDCF-0.25	0.25	Used	24.5	28.0	58.5	22.0	22.9	26.1
MDCF-0.50	0.50	Used	27.7	32.3	65.7	22.6	23.4	30.1
MDCF-0.75	0.75	Used	30.1	47.3	97.1	24.0	26.1	32.3

**Table 8:** Results of electrical resistance measurements.

Specimen ID	MWCNT Concentration (wt.%)	Use of MWCNT-Coated Films	Resistance (Ω)	
			At 40 mm Spacing	At 120 mm Spacing
PCC	0.00	None	88,110	98,590
MCF	0.75	Used	75,140	91,980
MD-0.25	0.25	None	87,030	98,260
MD-0.50	0.50	None	86,760	98,040
MD-0.75	0.75	None	85,250	97,520
MDCF-0.25	0.25	Used	1300	2220
MDCF-0.50	0.50	Used	320	820
MDCF-0.75	0.75	Used	180	590

## Data Availability

The original contributions presented in this study are included in the article. Further inquiries can be directed to the corresponding author.
